# Changes in serum neurofilament light chain levels following narrowband ultraviolet B phototherapy in clinically isolated syndrome

**DOI:** 10.1002/brb3.2494

**Published:** 2022-01-27

**Authors:** Marzena J. Fabis‐Pedrini, Jens Kuhle, Katherine M. A. Roberts, Stephanie Trend, Anderson P. Jones, Aleksandra Maceski, William M. Carroll, Robyn M. Lucas, Frank L. Mastaglia, Prue H. Hart, Allan G. Kermode

**Affiliations:** ^1^ Perron Institute for Neurological and Translational Science University of Western Australia Perth Australia; ^2^ Centre for Molecular Medicine and Innovative Therapeutics, Murdoch University Perth Australia; ^3^ Neurology Clinic and Policlinic MS Centre and Research Centre for Clinical Neuroimmunology and Neuroscience Basel University of Basel Basel Switzerland; ^4^ Telethon Kids Institute University of Western Australia Perth Australia; ^5^ National Centre for Epidemiology & Population Health Research School of Population Health Australian National University Canberra Australia; ^6^ Centre for Ophthalmology and Visual Science University of Western Australia Perth Australia; ^7^ Institute for Immunology and Infectious Disease Murdoch University Perth Australia

**Keywords:** clinically isolated syndrome, lesion volume, multiple sclerosis, neurofilament light chain, ultraviolet B

## Abstract

**Objective:**

To determine whether serum neurofilament light chain (sNfL) levels are suppressed in patients with the clinically isolated syndrome (CIS) following narrowband ultraviolet B phototherapy (UVB‐PT).

**Methods:**

sNfL levels were measured using a sensitive single‐molecule array assay at baseline and up to 12 months in 17 patients with CIS, 10 of whom received UVB‐PT, and were compared with healthy control (HC) and early relapsing remitting multiple sclerosis (RRMS) group. sNfL levels were correlated with magnetic resonance imaging total lesion volume (LV) determined using icobrain version 4.4.1 and with clinical outcomes.

**Results:**

Baseline median sNfL levels were significantly higher in the CIS (20.6 pg/mL, interquartile range [IQR] 13.7–161.4) and RRMS groups (36.6 pg/ml [IQR] 16.2–212.2) than in HC (10.7 pg/ml [IQR] 4.9–21.5) (*p* = .012 and *p* = .0002, respectively), and were strongly correlated with T2 and T1 LV at 12 months (*r* = .800; *p* = .014 and *r* = .833; *p* = .008, respectively) in the CIS group. Analysis of changes in sNfL levels over time in the CIS group showed a significant cumulative suppressive effect of UVB‐PT in the first 3 months (UVB‐PT −10.6% vs non‐UVB‐PT +58.3%; *p* = .04) following which the levels in the two groups converged and continued to fall.

**Conclusions:**

Our findings provide the basis for further studies to determine the utility of sNfL levels as a marker of neuro‐axonal damage in CIS and early MS and for assessing the efficacy of new therapeutic interventions such as UVB‐PT.

## Abstract 2

Serum neurofilament light chain (sNfL) levels were measured using a sensitive single‐molecule array assay at baseline and up to 12 months in 17 patients with the clinically isolated syndrome (CIS), 10 of whom received UVB‐phototherapy and seven did not receive UVB‐phototherapy. Longitudinal comparisons of sNfL levels in the UVB‐phototherapy treated versus non‐UVB‐phototherapy CIS participants using a mixed‐effects model showed that over the first 3 months there was a significant cumulative suppressive effect of UVB‐phototherapy.

## 1 INTRODUCTION

Previous studies in the experimental autoimmune encephalomyelitis (EAE), model of multiple sclerosis (MS) have demonstrated that ultraviolet radiation (UVR) can exert immunoregulatory effects independent of vitamin D pathways (Breuer et al., 2014; Irving et al., 2019). We have reported that narrowband UVB phototherapy (UVB‐PT) can decrease conversion rates from clinically isolated syndrome (CIS) to MS (Hart et al., 2018), making phototherapy a promising new treatment option. CIS is the first clinical manifestation in which a patient presents with symptoms and signs suggestive of an inflammatory demyelinating disorder of the CNS. The second clinically evident demyelinating attack separated in time and space from the first episode is known as a conversion from CIS to clinically definite MS (Polman et al., 2011). Prospective studies demonstrate that 60%–70% of CIS patients convert to MS within 20 years from onset (Miller et al., 2012). The mechanisms underlying the protective effect of UVB‐PT in CIS have not been investigated. Here, we monitored the longitudinal changes in serum neurofilament light chain (sNfL) levels as a measure of neuro‐axonal injury in CIS patients treated with an 8‐week course of UVB‐PT and correlated NfL levels with magnetic resonance imaging (MRI) lesion volumes (LV) and the risk of conversion to MS.

## 2 METHODS

### 2.1 Study participants

Seventeen individuals recruited within 3‐months of their first demyelinating event and meeting Paty A or Paty B MRI criteria (Paty et al., 1988) were included and were part of the PhoCIS study (Phototherapy for CIS) to assess the effects of narrowband UVB‐PT (311–312 nm) to prevent conversion from CIS to MS (Hart et al., 2018; Hart et al., 2017). Ten of the 17 were randomised to the phototherapy group and received UVB‐PT three times weekly for 8 weeks as previously described (Hart et al., 2018; Hart et al., 2017). Blood samples for NfL levels were taken at recruitment (baseline) and 1, 2, 3, 6, and 12 months. Briefly, peripheral venous blood was collected in Vacutainer SST tubes (Becton Dickinson, North Ryde, NSW, Australia) and serum separated by centrifugation at 800 × *g* for 10 min, then aliquoted and stared at −80°C until batch analysis. Although most follow‐up samples were collected from participants, there were a small number of samples not collected. The reasons for missing sample collection included withdrawal from the blood collection part of the study due to anxiety (n = 4 samples missed), when participants were not able to attend appointments (n = 7 samples missed), and relocation to another state following completion of phototherapy (n = 2 samples missed). A number of samples were also exhausted in previously published projects (n = 7 samples missed) (Hart et al., 2018; Trend et al., 2018; Trend et al., 2019; Trend et al., 2021) and one participant was lost to follow up after 1 week, and therefore could not be included in longitudinal analysis. In total, 82 blood samples were analyzed in UVB‐PT and non‐UVB‐PT. At baseline, all CIS participants were drug naïve. Individuals who had short courses of intravenous corticosteroids (two in the UVB‐PT group and three in the non‐UVB‐PT group), were required to have a 1‐month wash‐out period before blood collection. Two comparison groups provided single blood samples: nine patients with a diagnosis of RRMS according to the McDonald 2010 criteria (Polman et al., 2011) and recent clinical symptoms, and 10 healthy controls (HC) with no history of autoimmune disease (Table 1). Participants with serum 25(OH)D levels < 80 nmol/L at enrolment were advised to take oral vitamin D supplements (up to 5000 IU/day).

**TABLE 1:** Characteristics of study groups

Characteristic	HC (n = 10)	UVB‐PT (n = 10)	Non‐UVB‐PT (n = 7)	RRMS (n = 9)
Age at study entry, y, mean ± SD	35.6 ± 8.4	39.0 ± 6.1	40.0 ± 10.3	36.2 ± 12.9
Sex, n (%)				
Female	8 (80)	4 (40)	7 (100)	7 (78)
Male	2 (20)	6 (60)	–	2 (22)
Type of onset, n (%)				
Monofocal	NA	10 (100)	7 (100)	9 (100)
Brainstem		1 (10)	–	1 (11)
Optic nerve		6 (60)	4 (57)	2 (22)
Spinal cord		3 (30)	1 (14)	6 (67)
Other		–	2 (29)	–
EDSS, median (min – max)	NA	1.0 (0 – 2.0)	1.5 (0 – 3.0)	2.0 (0 – 3.0)
Serum 25(OH)D levels, nmol/L, median, (IQR)	66.3 (47.2 – 71.5)	92.5 (55.3 – 110.6)	79.3 (66.3 – 117.6)	74.7 (62.1 – 83.3)
Serum NfL levels, pg/mL, median, (IQR)	10.7 (4.9 – 21.5)	20.7 (15.4 – 29.6)	20.1 (14.1 – 50.5)	36.6 (16.2 – 212.2)

Abbreviations: EDSS, Expanded Disability Status Scale; HC, healthy controls; IQR, interquartile range; RRMS, relapsing remitting multiple sclerosis; UVB‐PT, ultraviolet B phototherapy.

### 2.2 Serum NfL assay

Serum NfL concentration was measured using a single‐molecule array (SIMOA^®^) platform at the University Hospital Basel as previously described (Disanto et al., 2017). Samples and calibrators were measured in duplicate. Seven non‐zero calibrators were measured in each run. The mean intra‐assay coefficient of variation of duplicated determinations for concentration was 6.2%. Measurements were performed on coded samples. All laboratory personnel remained blinded to clinical data and diagnosis.

### 2.3 MRI acquisition and analysis

Three‐dimensional fluid‐attenuated inversion recovery (FLAIR) (1 mm), T1‐, T2‐weighted, and axial T1‐weighted post‐gadolinium sequences were acquired on a 3‐Tesla magnet. Brain volumetry was determined by submitting the DICOM data (3D FLAIR and 3D T1 MP‐RAGE images) to the Icometrix portal (Leuven, Belgium; Chicago, USA). Total lesion volume (LV) was computed using icobrain version 4.4.1.

### 2.4 Statistical analysis

Analyses were performed using GraphPad Prism v8 (GraphPad Software, La Jolla, CA). Kruskal–Wallis analysis of variance with Dunn's post hoc correction was used to compare sNfL levels between groups. Correlations between sNfL levels and clinical or MRI outcomes were calculated with Spearman's test. Levels of sNfL in UVB‐PT and non‐UVB‐PT groups were analyzed as percentage change from baseline, using the calculation ((value‐baseline)/baseline) × 100. The mixed‐effects model with fixed and random effects with the Geisser‐Greenhouse correction (Tukey's multiple comparison test) was used to evaluate the longitudinal effects of phototherapy on sNfL levels across the follow‐up period, controlling for baseline levels as a covariable. Mann–Whitney *U* test was used to compare results between baseline and 3 months timepoints in both UVB‐PT and non‐UVB‐PT groups. All CIS participants with at least one additional sample collected after baseline were included in the analysis. A nominal *p*‐value of < .05 was considered statistically significant.

### 2.5 Ethics approvals

The study protocol was approved by the Bellberry Human Ethics Committee (2014‐02‐082) and Sir Charles Gairdner Hospital Human Research Ethics Committee (2006‐073). The PhoCIS trial was registered with the Australian and New Zealand Clinical Trials Registry (ACTRN 12614000185662, registered 19/02/2014). All participants provided written informed consent. This study conformed to the Strengthening the Reporting of Observational Studies in Epidemiology (STROBE) reporting guideline.

## 3 RESULTS

All 17 CIS patients were drug‐naïve at baseline and for the first 3 months of the follow‐up period, which included the 8‐week course of UVB‐PT. All participants in the non‐UVB‐PT group met the criteria for diagnosis of MS by 12 months compared to 70% in the UVB‐PT group (one patient in the non‐UVB‐PT group was lost to follow‐up). During this period, five participants in the UVB‐PT group and four participants in the non‐UVB‐PT group were commenced on disease‐modifying therapy (DMT; dimethyl fumarate, natalizumab, or fingolimod).

### 3.1 Serum NfL levels

Serum NfL levels at baseline were significantly elevated in both the CIS and RRMS groups compared to HCs. Median sNfL levels in CIS and RRMS patients were 20.6 (interquartile range [IQR] 13.7–161.4) pg/ml and 36.6 ([IQR] 16.2–212.2) pg/ml, respectively, and were significantly higher (*p* = .012 and *p* = .0002, respectively) than in HC (10.7 ([IQR] 4.9–21.5) pg/ml, but sNfL levels in the CIS and RRMS groups were not significantly different. There was no significant difference in sNfL levels at baseline between UVB‐PT and non‐UVB‐PT CIS groups (20.7 and 20.1 pg/ml, respectively; *p* = .494), or correlations with age, EDSS score, or serum 25(OH)D levels. Baseline sNfL levels were not significantly correlated with the risk of conversion to MS during the 12‐month study period (*p* = .661).

### 3.2 Effect of UVB phototherapy on NfL levels

We have previously examined the effect of narrowband UVB‐PT on patients with CIS and their conversion to MS (Hart et al., 2018). The reduced conversion rate in the UVB‐treated participants let us formulate a question of whether there is a protective association of UVB‐PT exposure on MRI measures of neurodegeneration in MS, expressed as changes in the sNfL levels.

**FIGURE 1:**
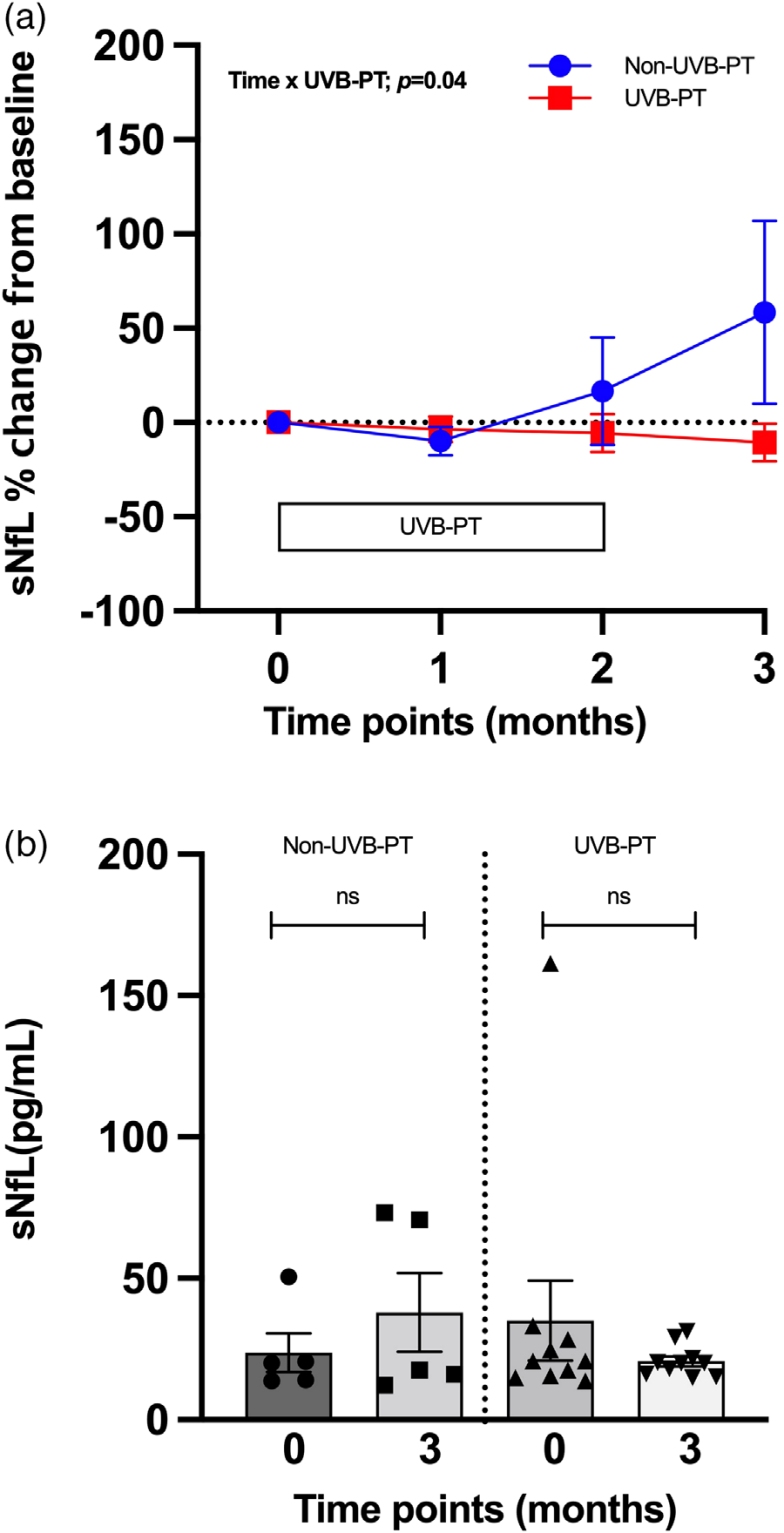
Effect of UVB Phototherapy on Serum NfL Levels. (A) The percentage change from baseline sNfL levels, measures in individuals from non‐phototherapy (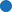) (baseline, n = 5; 1 month, n = 2; 2 months, n = 3; 3 months, n = 5) and phototherapy (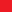) (baseline, n = 10; 1 month, n = 9; 2 months, n = 9; 3 months, n = 10) groups. The group means and SEM are shown. Longitudinal effects of phototherapy on sNfL levels were analyzed by fitting a mixed‐effects model. *p = .04 was observed for cumulative effect of time × phototherapy over first 3 months. (B) Differences between sNfL levels at baseline (0) and at 3 months (3) in UVB phototherapy and non‐UVB phototherapy groups. Box with individual values is shown as a mean ± SEM. Significance was tested with the Mann–Whitney *U* test

Evaluating the effect of narrowband UVB phototherapy on the sNfL levels in CIS patients, we analyzed the percentage change from the baseline. Using a mixed‐effects model analysis for repeated measures, we have observed a significant cumulative effect of time and phototherapy (*p *= .04, F = 3.12). At the 3‐month time point, in the UVB‐PT group, the median sNfL level was lower by 10.6%, whereas in the non‐UVB‐PT group, it was higher by 58.3% (Figure 1A). The individual values are shown in Figure 1B. The 3‐month time point is considered important to evaluate the effect of UVB‐PT on sNfL levels since the UVB treatment finished after 8 weeks and all patients were still DMT‐free.

### 3.3 Correlations between sNfL levels and brain lesion volume in CIS

At baseline, sNfL levels in the CIS group were marginally correlated with T2‐LV (*p* = .058), but not T1‐LV (*p* = .108). Baseline sNfL levels were strongly correlated with both T2‐LV and T1‐LV at 12 months (*r* = .800; *p* = .014 and *r* = .833; *p* = .008, respectively). There were no statistically significant differences between UVB‐PT and non‐UVB‐PT groups at baseline in T2‐LV (2.29 (0.71–10) mL and 2.03 (1.42–3.27) ml, respectively, *p* = .604), however, there was a correlation between baseline sNfL levels and T1‐LV in the UVB‐PT group at 12 months (*p *= .02).

## 4 DISCUSSION

We monitored sNfL levels following UVB‐PT in a CIS cohort and investigated correlations with MRI LV and clinical outcomes. Our results showed that sNfL levels were significantly elevated (at baseline) in the CIS cohort, and in a control RRMS group, compared to HC, in line with the findings of other studies corroborating that circulating NfL levels are associated with neuro‐axonal damage and are a marker for disease activity (Bittner et al., 2020; Disanto et al., 2017; Khalil et al., 2018; Kuhle et al., 2019). An initial suppression of sNfL levels was apparent in the UVB‐PT group during the first 3 months, during which all patients were drug‐naïve, suggesting that UVB‐PT may have had a beneficial effect in reducing the severity of the neuro‐axonal injury.

The CIS patients selected for the UVB‐PT study all met the Paty A or Paty B criteria (i.e., the presence of at least four T2 lesions greater than 3 mm, or at least three such T2 lesions, one being periventricular) and were therefore at high risk of conversion to MS. A comparative follow‐up showed that the rate of conversion to MS in the subsequent 9 months was lower in the UVB‐PT group (70% vs 100% in the untreated group), as was the number of patients who commenced DMT during that period. The convergence of sNfL levels in the two CIS groups after the first 3 months may have been due to the effects of DMT, as a decrease in sNfL levels has been linked to DMT in larger MS cohorts (Kuhle et al., 2019; Piehl et al., 2018). However, the possibility of an ongoing effect of UVB‐PT cannot be excluded in the UVB‐PT treated group and may have been complemented by DMT in patients who received DMT. We found a correlation between baseline sNfL levels and T2‐ and T1‐LV in CIS patients, in keeping with the findings of previous studies that showed an association between plasma or serum NfL and MRI measures (Dalla Costa et al., 2019; Kuhle et al., 2019).

Our findings point to a temporary protective effect of UVB‐PT in a group of CIS patients at high risk of progressing to MS. However, the study had limitations because of the small patient numbers and the findings require confirmation in a larger longitudinal patient cohort.

## CONFLICT OF INTEREST

M.J. Fabis‐Pedrini has received travel sponsorship from Merck Serono Australia. J. Kuhle received speaker fees, research support, travel support, and/or served on advisory boards by Swiss MS Society, Swiss National Research Foundation (320030_189140/1), University of Basel, Progressive MS Alliance, Bayer, Biogen, Celgene, Merck, Novartis, Roche, Sanofi. W.M. Carroll has received speaker honoraria and scientific advisory board fees from Bayer, BioCSL, Biogen‐Idec, Lgpharma, Merck, Novartis, Roche, Sanofi‐Aventis, Sanofi‐Genzyme, Teva. A.G. Kermode has received speaker honoraria and scientific advisory board fees from Bayer, BioCSL, Biogen‐Idec, Lgpharma, Merck, Novartis, Roche, Sanofi‐Aventis, Sanofi‐Genzyme, Teva, NeuroScientific Biopharmaceuticals, and Mitsubishi Tanabe. A. Maceski, K.M.A. Roberts, A.P. Jones, S. Trend, R.M. Lucas, F.L. Mastaglia, and P.H. Hart report no disclosures.

### PEER REVIEW

The peer review history for this article is available at https://publons.com/publon/10.1002/brb3.2494

## Data Availability

Anonymised data will be shared by request from any qualified investigator.
